# Investigations of Different Ion Intercalations on the Performance of FBG Hydrogen Sensors Based on Pt/MoO_3_

**DOI:** 10.3390/s19214775

**Published:** 2019-11-03

**Authors:** Gaopeng Wang, Shiwen Yang, Jixiang Dai, Yutang Dai, Tong Zou, Johannes Roths, Minghong Yang

**Affiliations:** 1 National Engineering Laboratory for Fiber Optic Sensing Technology, Wuhan University of Technology, Wuhan 430070, China; w32136@whut.edu.cn (G.W.); sherkyyang@163.com (S.Y.); djx409081947@163.com (J.D.); daiyt6688@whut.edu.cn (Y.D.); 2 Institute of Seismology, China Earthquake Administration, Wuhan 430071, China; Tong.zou@eqhb.gov.cn; 3 Munich University of Applied Sciences, Lothstr. 34, D-80335 Munich, Germany; j.roths@hm.edu

**Keywords:** α-MoO_3_, ion intercalation, hydrogen sensors, repeatability, optimization

## Abstract

α-MoO_3_ has been used as a hydrogen sensing material due to its excellent properties and unique crystalline layer structure. However, the low repeatability of α-MoO_3_ based hydrogen sensor restricts its practical application. In this paper, the effect of intercalated ion species and the amount in α-MoO_3_ is experimentally investigated and discussed. It is concluded that the repeatability of the sensor depends on the radius of intercalated ions and amount of ionic bonds. The optimal ion species is Na^+^ and the optimal amount of precursor is 1 mmol.

## 1. Introduction

Hydrogen has been considered as the most promising energy to substitute fossil fuels due to its clean and reusable characteristics. However, hydrogen is prone to leak during production, transportation, and utilization. Additionally, it will explode in air when the concentration lies in the range of 4–75%. Among varieties of hydrogen sensors, fiber Bragg grating (FBG) hydrogen sensors have drawn much research interest for its electrical-free characteristic and the ability of quasi-distributed measurement [1,2,3]. One of the most urgent problems in the application of the FBG hydrogen sensors is the low repeatability of the sensing material [4].

MoO_3_ has been recognized as a multifunctional material for its special crystal structure and unique electrical properties. Numerous researches have demonstrated its potential use as hydrogen sensing material [5,6,7,8,9,10]. However, the layer structure of α-MoO_3_ has the potential to collapse after a long time reaction with hydrogen. In our previous work [11], an ion intercalation method was proposed to improve the repeatability of the sensor. It was found that the Na ion intercalated with MoO_3_ exhibits the best performance by improving the sensor’s repeatability by an order of magnitude. The improved repeatability was ascribed to the stabilized layer structure by ion intercalation. However, the layer distance (1.4 nm) of α-MoO_3_ is broad enough for ions such as Li^+^ and K^+^ to be intercalated into the layer structure. The intercalated ions will stabilize the layer structure by forming ion bonds. As the bond force varies from ion species and the amount of the intercalated ions, these two factors may also influence the sensing performance of the intercalated MoO_3_.

In this paper, we proposed an ion intercalation method to improve the repeatability of FBG hydrogen sensors based on α-MoO_3_. The effect of ion species and the amount of the intercalated ions on the sensitivity and repeatability of the sensors were systematically investigated and discussed. The optimal ion species and amount of intercalated ions were obtained by numerous experiments.

## 2. Experimental

### 2.1. Synthesis of the Sensing Material

Based on our previous work [11], Pt/MoO_3_ was synthesized by a hydrothermal method stepped by a sintering process. In detail, 2 g of molybdenum powder (Shanghai Macklin Biochemical Co., Ltd., Shanghai, China) was slowly added into 45 mL H_2_O_2_ (30%) (Sinopharm Chemical Reagent Co., Ltd., Shanghai, China). Then the mixture was continuously stirred overnight and transferred into a 50 mL Teflon-lined stainless steel autoclave, which was then heated at 180 °C for 7 h. The obtained precipitation was separated by centrifugation, and MoO_3_ nanobelts were obtained after the precipitation was dried at 60 °C for 12 h. After that, 0.14 g as-prepared MoO_3_ and 0.084 g acetylacetone platinum (Pt(acac)_2_) were mixed, ground, and sintered at 400 °C for 5 h to obtain Pt/MoO_3_.

Ion intercalated MoO_3_ was prepared to improve the performance of the sensors. In this paper, ion intercalated MoO_3_ is prepared by a two-step hydrothermal method. The first hydrothermal process has been illustrated above. Then 0.36 g as-prepared MoO_3_ nanobelts, 0.3 g polyethylene-glycol (PEG) (Sinopharm Chemical Reagent Co., Ltd.), and 1 mmol MCl (M: Li, Na, and K) (Sinopharm Chemical Reagent Co., Ltd.) were dissolved in 60 mL of deionized water under stirring for 2 h. Then the solution was transferred into a Teflon-lined stainless steel autoclave and then kept at 180 °C for 2 days. Ion intercalated MoO_3_ was obtained after the precipitation was washed and dried for 12 h. The amount of the intercalated ions was controlled by changing the amount of MCl to 1, 2, and 5 mmol. The obtained ion intercalated MoO_3_ was then mixed with Pt(acac)_2_ according to the ratio as mentioned above. Then, the mixture was sintered at 400 °C for 5 h, the obtained powder was named as M(*x*)-Pt/MoO_3_, where M refers to ion species such as Li, Na, and K, *x* is the amount of MCl added in the hydrothermal process.

### 2.2. Fabrication of the Sensor and Sensing Performance Tests

The sensor device was fabricated by uniformly coating the as-synthesized Pt/MoO_3_ powder on a fiber with two standard FBGs which had a separation of 2 cm. Firstly, the FBGs were fixed on a customized quartz substrate with a groove by epoxy glue (353ND, Epoxy Technology Inc., Billerica, MA, USA), then 0.03 g obtained Pt/MoO_3_ was mixed with 15 μL de-ionized water to form a slurry. After that, the slurry was transformed into the groove and heated by a heat gun. Finally, the coating was formed on the surface of one FBG. Another FBG was bare in the air for temperature compensation.

**Figure 1:**
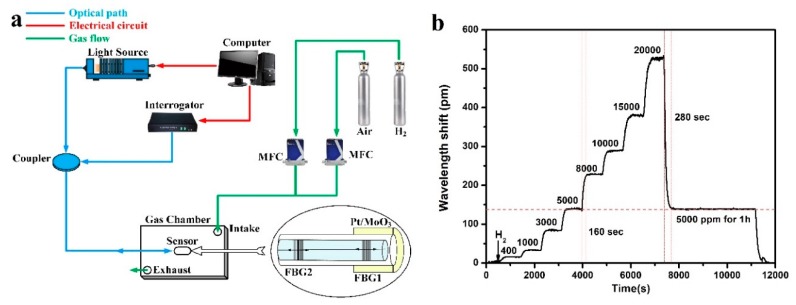
(**a**) Configuration of fiber Bragg grating (FBG) hydrogen sensing system. (**b**) Hydrogen response of Pt/MoO_3_.

The sensing performance tests were conducted on the system (Figure 1a). The FBG1 was coated with the sensing material to detect hydrogen and FBG2 was introduced as reference for temperature compensation. The center wavelength of FBG changes due to the heat released by an exothermic reaction between the sensing material and hydrogen. Hydrogen sensing tests were conducted on room temperature under various hydrogen concentrations. In order to explore the detection limit and the stability of the sensor, the sensor based on Pt/MoO_3_ was tested under hydrogen concentration of 400, 1000, 3000, 5000, 8000, 10,000, 15,000, and 20,000 parts per million (ppm) in volume fraction in air, respectively. Each stage of hydrogen concentration was maintained for 10 min, controlled by two mass flow controllers (MFC). After that, the hydrogen concentration was decreased to 5000 ppm and maintained for 1 h.

To investigate the relationship between the amount of intercalated ions and the sensors’ repeatability, the sensors were tested under a hydrogen concentration of 15,000 ppm for 1 h, synthetic air was injected for 10 min in the chamber to eliminate the humidity effect. In order to better understand the sensor’s repeatability in a dynamic change of hydrogen concentrations, the following experiment was conducted. The fabricated sensors were exposed at a hydrogen concentration of 1500, 3000, 6000, 9000, 12,000, and 15,000 ppm successively for 10 min under each concentration. Then the sensors were flushed by synthetic air for 10 min, this will exhaust the hydrogen and maintain the sensors to its initial state. The wavelength shifts of the sensor at each concentration were recorded and the data were nonlinearly fitted according to the relationship used in our previous study [12,13]. This cycling test was conducted once a day and repeated 50 times in 2 months.

### 2.3. Materials Characterizations

The X-ray diffraction (XRD) measurement was performed to investigate the crystal information using a Bruker D8 Advance X-ray diffractometer with Cu Kα X-ray source operating at 40 kV. The elements amount in the samples was characterized by the inductively coupled plasma (ICP) test using the Perkin Elmer Optima 4300DV spectrometer.

## 3. Results and Discussion

As the stability of the layer structure of MoO_3_ is affected by the ion species and amount, 10 samples with different intercalated ions were prepared and tested during all of the experiments. The detailed information of the ten samples is shown in Table 1 where 1 mmol, 2 mmol, and 5 mmol refer to the amount of the MCl (M: Li, Na, and K) in the precursor. As the experiment results in our previous study showed that the sensors exhibited worse performance when the amount of the precursor exceeded 5 mmol [11], the precursor amount was controlled under 5 mmol in this work.

The sensors based on ion intercalated MoO_3_ also exhibit good stability in their first test. The testing result is shown in Figure 1b. It is found that the sensor exhibits an obvious and sharp response towards various hydrogen concentration and the detection limit is 400 ppm. Additionally, the sensor responds immediately as hydrogen concentration changes, the response time is usually 3 min, while the recovery time is a little longer. Moreover, the sensor shows good stability when it is exposed in a hydrogen concentration for 1 h, the fluctuation of the wavelength shifts during this period of time is within 2 pm. In addition, in both of the tests at 5000 ppm, the sensor has the same response, i.e., 139 ± 3 and 139 ± 1 pm, respectively.

**Figure 2:**
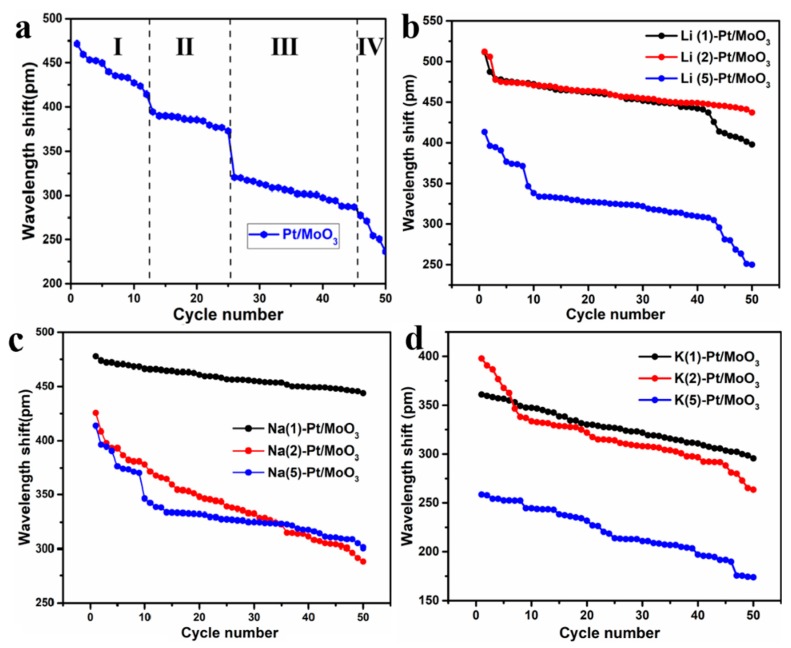
Repeatability of (**a**) Pt/MoO_3_, (**b**) Li^+^ intercalated Pt/MoO_3_, (**c**) Na^+^ intercalated Pt/MoO_3_ and (**d**) K^+^ intercalated Pt/MoO_3_.

Figure 2 shows the testing results of the sensors. Figure 2a shows the sensor exhibited an obvious degradation phenomenon which is caused by the structural collapse of MoO_3_ and agglomeration of Pt particles [11]. Pt/MoO_3_ is reduced by hydrogen and produced both water and heat. The wavelength shifts of Pt/MoO_3_ decreased from 475 pm to 236 pm in the 50 tests. All the tests can be divided into four stages as three sharp declines occurred. It can be speculated that individual MoO_3_ molecules will be degraded during each stage. Then, quantitative changes lead to qualitative change, whole MoO_3_ layer degradation happens finally. For ion intercalated samples, the degradation phenomenon was obviously slowed down. In Figure 2b, it can be found that the Li^+^ intercalated samples still exhibit a degradation phenomenon but the rate of the degradation was significantly reduced. Li(1)-Pt/MoO_3_ and Li(2)-Pt/MoO_3_ exhibited almost the same response in the first 40 tests, a sharp decline in wavelength shift about 25 pm occurred in the second test for both two samples. In the last ten tests, the degradation rate of Li(1)-Pt/MoO_3_ increased while it was reduced for Li(2)-Pt/MoO_3_, which means that Li(2)-Pt/MoO_3_ exhibited a better repeatability than Li(1)-Pt/MoO_3_. In addition, Li(5)-Pt/MoO_3_ exhibited the worst repeatability and the sensitivity was much lower than the other two samples. The testing results of Na^+^ intercalated samples were shown in Figure 2c. Similar to Li^+^ intercalated samples, all of the three samples degraded during the tests. The degradation rate is much lower than that of Pt/MoO_3_, which means Na^+^ intercalation can improve the repeatability of the sensors. However, the optimal amount of NaCl added in a hydrothermal process lies in 1 mmol and the improvement of the repeatability reduced with the increasing amount of NaCl. Figure 2d illustrates the cycling test results of K^+^ intercalated MoO_3_. Similar to Li^+^ and Na^+^ intercalated samples, K^+^ intercalated samples exhibited better repeatability than Pt/MoO_3_. K(1)-Pt/MoO_3_ did not show a sharp drop in wavelength drift during the 50 tests, and its wavelength shift decreased 1–2 pm each time during the tests. During the 1–8 tests of K(2)-Pt/MoO_3_, the wavelength shift decreased about 6–7 pm each time, the decreased rate slowed down to about 1–2 pm each time in the 9–45 tests. During the 46–50 tests, the wavelength shift decreased faster at a rate of about 3–5 pm each time. The wavelength shifts of K(5)-Pt/MoO_3_ stepped down gradually during the 50 tests, the wavelength shift in each step slowly decreased, and the falling speed was 1–2 pm each time. K(5)-Pt/MoO_3_ showed much lower sensitivity than K(1)-Pt/MoO_3_ and K(2)-Pt/MoO_3_. Combining the synthesis process, excess intercalated K^+^ with a relatively large ionic radius may exfoliate the layer structure and result in lower sensitivity to hydrogen.

In summary, ion intercalated Pt/MoO_3_ shows better repeatability than pristine Pt/MoO_3_. The improvement in the sensor’s repeatability is affected by the amount of intercalated ions. The optimal amount of MCl lies in 2 mmol, 1 mmol, and 1 mmol for Li^+^, Na^+^, and K^+^, respectively. When the MCl amount exceeds the optimal value, the repeatability will decrease with the increase of the MCl amount.

**Figure 3:**
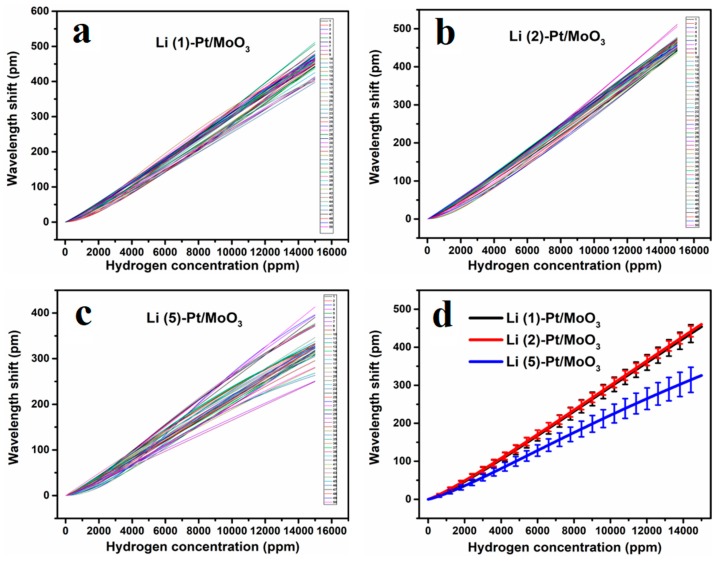
Repeatability of (**a**) Li(1)-Pt/MoO_3_, (**b**) Li(2)-Pt/MoO_3_, (**c**) Li(5)-Pt/MoO_3_ and (**d**) error analyses of the three samples.

Figure 3 shows the cycling test results of Li^+^ intercalated Pt/MoO_3_. It can be concluded that the growth rate of each fitting curve increases with the increase of hydrogen concentration, indicating that the sensitivity of the sensor becomes larger as the hydrogen concentration increases. According to the hydrogen sensing principle of Pt/MoO_3_ [8], when the hydrogen concentration is low, the hydrogen sensing reaction rate is controlled by the diffusion process of hydrogen. A lower hydrogen concentration results in a smaller hydrogen concentration gradient inside and outside of the sensitive material, which means a lower reaction rate [11]. The heat exchange between the hydrogen sensitive material and the FBG is quickly balanced to stabilize the FBG’s temperature at a relatively low value. Therefore, at low concentrations, the sensitivity of the sensor is low; when the hydrogen concentration is high, the reaction is controlled by the catalytic rate. The higher hydrogen concentration gradient allows hydrogen to be quickly adsorbed onto the surface of the hydrogen sensitive material. The Mo^5+^ generated by the hydrogen reduction process made MoO_3_ have a higher affinity to hydrogen, which accelerated the adsorption of hydrogen by sensitive materials. In addition, the heat generated by the reaction increases the catalytic activity of the catalyst, these two factors result in high sensitivity in high hydrogen concentration. The dispersion degree of the 50 fitted lines reflects the repeatability of the sensors. The concentrated lines of Li(2)-Pt/MoO_3_ indicate the best repeatability and Li(5)-Pt/MoO_3_ exhibits the worst repeatability. The error analyses in Figure 3d provide more accurate information about the sensor’s repeatability. It can be concluded that the relative errors of Li(1)-Pt/MoO_3_, Li(2)-Pt/MoO_3_, and Li(5)-Pt/MoO_3_ are ±5.8%, ±3.0%, and ±10.5%, respectively. Additionally, the fitting curve of Li(5)-Pt/MoO_3_ in Figure 3d is always under those of Li(1)-Pt/MoO_3_ and Li(2)-Pt/MoO_3_, which means the sensitivity of Li(5)-Pt/MoO_3_ is lower than Li(1)-Pt/MoO_3_ and Li(2)-Pt/MoO_3_.

**Figure 4:**
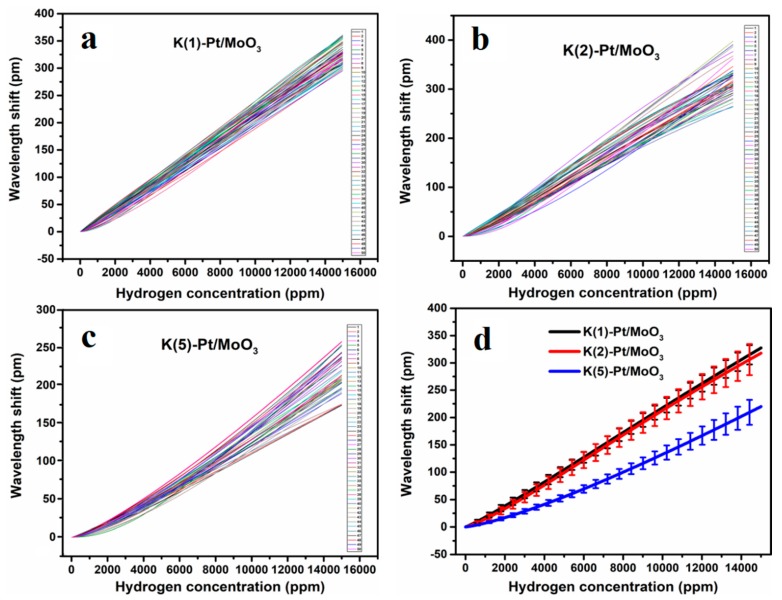
Repeatability of (**a**) K(1)-Pt/MoO_3_, (**b**) K(2)-Pt/MoO_3_, (**c**) K(5)-Pt/MoO_3_ and (**d**) error analyses of the three samples.

**Figure 5:**
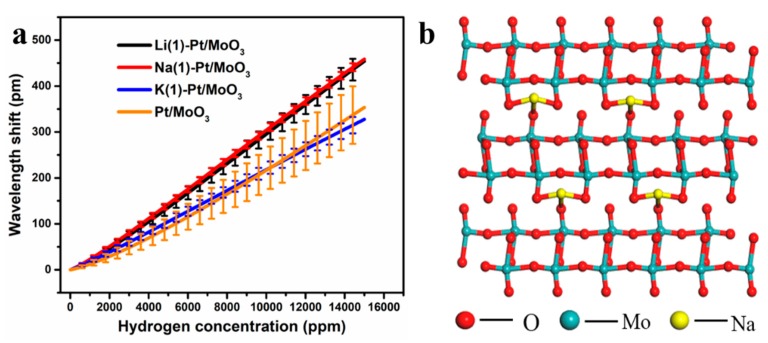
(**a**) Error analyses of Pt/MoO_3_, Li(1)-Pt/MoO_3_, Na(1)-Pt/MoO_3_, and K(1)-Pt/MoO_3_, (**b**) Schematic illustration of the crystalline structure of ion intercalated MoO_3_.

Figure 4 illustrates the cycling test results of K^+^ intercalated Pt/MoO_3_, the relative errors of K(1)-Pt/MoO_3_, K(2)-Pt/MoO_3_, and K(5)-Pt/MoO_3_ are ±5.5%, ±9.5%, and ±10.9%, respectively. With the increasing amount of KCl, the repeatability and sensitivity of the sensors decrease, which is similar to Na^+^ intercalated samples. Based on the testing results and our previous study [11], the error analyses results of Pt/MoO_3_ and ion intercalated samples when the precursor amount is 1 mmol are shown in Figure 5. It is found that the pristine Pt/MoO_3_ exhibited the worst repeatability with a relative error of ±18%, the repeatability of the sensor can be significantly improved by ion intercalation. When the amount of MCl lies in 1 mmol, Na^+^ intercalation improves the sensor’s repeatability the most, Li^+^ ranges the second and K^+^ has the lowest improvement in repeatability. This may be ascribed to the binding force between the layer structure.

Figure 5b illustrates the proposed mechanism of improving performance by ion intercalation [14]. Three kinds of oxygen atoms on α-MoO_3_ can be named as unshared oxygen, edge-sharing oxygen, and corner-sharing oxygen. The unshared oxygen atoms with non-bonding electrons form only one covalent bond, thus they will bond with the intercalated ions. The ions were intercalated into the double-layer structure of α-MoO_3_ by a hydrothermal method, some of the intercalated ions will bond with the unshared oxygen atoms, transforming the van der Waals force into ionic bonds [11], which is much stronger to maintain the layer structure during the reaction with hydrogen. However, the layer structure’s stability depends on the amount of M-O (M: Li, Na, and K) bond and the bonding force of the M-O bond. Ranked in order of the binding force, the Li-O bond is stronger than Na-O and K-O ranks at the bottom among them. So according to this assumption, the repeatability of the samples should be ranked as: Li(1)-Pt/MoO_3_ > Na(1)-Pt/MoO_3_ > K(1)-Pt/MoO_3_ > Pt/MoO_3_, which is inconsistent with the experiment. It should be mentioned that the amount of ionic bonds formed in the layer structure is not equal to the MCl amount in the precursor. The actual ion amount in the layer structure is dominated by the reaction conditions and the species of reactants. The element contents of the ion intercalated samples were analyzed by ICP and the results are shown in Table 2.

It can be concluded that the actual ion amount in the intercalated samples varies from ion species, the molar ratio of M/Mo of Li(1)-Pt/MoO_3_, Na(1)-Pt/MoO_3_, and K(1)-Pt/MoO_3_. The amount of Li^+^ intercalated in the sample is much lower than Na^+^. Although the Li–O bond is stronger than the Na-O bond, the lower concentration results in fewer ionic bonds. Combining these two factors, the binding force between the layer structure of Li(1)-Pt/MoO_3_ is lower than that of Na(1)-Pt/MoO_3_. The relative low stability of the Li(1)-Pt/MoO_3_’s layer structure is easier to collapse during the reaction with hydrogen and results in lower repeatability, which has been confirmed by the experiments. As for the K^+^ intercalated samples, the relatively large ionic radius and high concentration may expand the layer distance and results in lower stability of the layer structure compared to the other samples.

**Figure 6:**
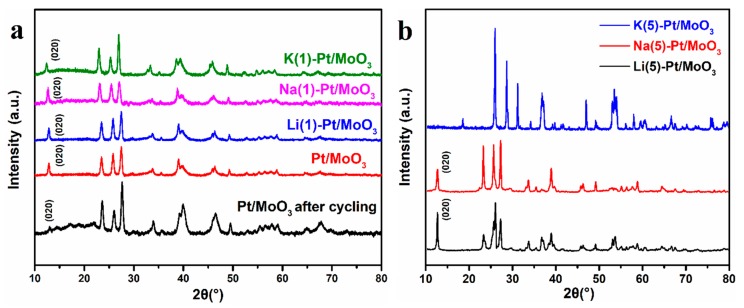
(**a**)XRD patterns of Pt/MoO_3_ after cycling, Pt/MoO_3_, Li(1)-Pt/MoO_3_, Na(1)-Pt/MoO_3,_ and K(1)-Pt/MoO_3_. (**b**) XRD patterns of Li(5)-Pt/MoO_3_, Na(5)-Pt/MoO_3_, and K(5)-Pt/MoO_3_.

To investigate the structure change during the ion intercalated process, XRD patterns of the samples were collected and the results are shown in Figure 6. Figure 6a exhibits the XRD patterns of Pt/MoO_3_ after cycling, Pt/MoO_3_, Li(1)-Pt/MoO_3_, Na(1)-Pt/MoO_3_, and K(1)-Pt/MoO_3_. The diffraction peaks of the XRD pattern for all of the samples can be indexed to be orthorhombic with lattice constants of a=3.962 Å, b=13.85 Å, c=3.697 Å (JCPDSNo.05-0508) and the cubic phase of Pt (JCPDS No.00-004-0802), which means the intercalation process did not change the major phase of MoO_3_. However, the layer structure diffraction peaks corresponding to the (020) plane varies from each sample. The (020) diffraction peak of Pt/MoO_3_, Li(1)-Pt/MoO_3_, Na(1)-Pt/MoO_3,_ and K(1)-Pt/MoO_3_ are 12.94°, 12.84°, 12.69°, and 12.39°, respectively. For the sample Pt/MoO_3_, diffraction peak at 12.94° disappeared after the cycling test, which corresponds to the (020) plane. It indicates that the layer structure collapses after the cycling test. The layer structure diffraction peaks show a small shift towards low degree with the increasing of ion radius, which means the distance between the interlayer was expanded after ion intercalation. Combining with the ICP results, high concentration of intercalated K^+^ did not result in a stronger structure stability than the Li^+^ and Na^+^ intercalated samples. In contrast, the higher concentration of K^+^ with a large radius expanded the interlayer distance. Additionally, the relatively weak K–O bond didn’t provide enough binding force with the interlayer compared to the other samples, resulting in lower repeatability of the sensor.

In addition, it is found in the experiments that when the amount of MCl exceeds 5 mmol, the average wavelength shifts decreased sharply whatever ion species [11]. The average wavelength shifts of Li(5)-Pt/MoO_3_, Na(5)-Pt/MoO_3,_ and K(5)-Pt/MoO_3_ at a hydrogen concentration of 15,000 ppm are 325, 334 and 219 pm. K(5)-Pt/MoO_3_ exhibited a much lower sensitivity than Li(5)-Pt/MoO_3_ and Na(5)-Pt/MoO_3_. Combining with the analyses above, K^+^ with a large radius will expand the layer structure and may change the crystalline structure of MoO_3_. XRD analyses were conducted to investigate the potential structure change of MoO_3_ and the results are shown in Figure 6b. XRD patterns of Li(5)-Pt/MoO_3_ and Na(5)-Pt/MoO_3_ are almost the same and they show typical diffraction peaks of α-MoO_3_, which means the ion intercalation of Li^+^ and Na^+^ did not change the major phase of MoO_3_. However, K(5)-Pt/MoO_3_ exhibited different diffraction peaks compared to the others, it can be concluded that the sample mainly consists of a mixture of MoO_2_ and MoO_3_, which means some of MoO_3_ in the sample was reduced to MoO_2_, which is similar to the previous work [14]. As the affinity to hydrogen of MoO_2_ is much lower than MoO_3_, the sensor exhibited extremely lower sensitivity.

## 4. Conclusions

In this paper, the effects of ion species and the amount of the precursor to the performance of FBG hydrogen sensors were investigated in detail. It is demonstrated that the instability of the layer structure of MoO_3_ results in low repeatability. Ion intercalation can improve the repeatability of the sensor by reinforcing the layer structure and the improvement varies depending on the ion species and amount of ionic bonds. The optimal ion species is Na^+^ and the optimal amount of precursor is 1 mmol under current synthesis condition. Some of the MoO_3_ may be reduced to MoO_2_ during the synthesis process, which results in a decline in the repeatability of the sensor. This work demonstrates the influence of the intercalated ion species and amount used to the repeatability of the sensors based on Pt/MoO_3_ as shown in numerous experiments, and it provides an instructional method in preparing appropriate sensing material used in FBG hydrogen sensors for practical use.

## Figures and Tables

**Table 1:** Samples used in the experiment.

Species	1 mmol	2 mmol	5 mmol	0 mmol
Li	Li(1)-Pt/MoO_3_	Li(2)-Pt/MoO_3_	Li(5)-Pt/MoO_3_	/
Na	Na(1)-Pt/MoO_3_	Na(2)-Pt/MoO_3_	Na(5)-Pt/MoO_3_	/
K	K(1)-Pt/MoO_3_	K(2)-Pt/MoO_3_	K(5)-Pt/MoO_3_	/
/	/	/	/	Pt/MoO_3_

**Table 2:** Inductively coupled plasma (ICP) results of the ion intercalated MoO_3._

Sample	M (Li, Na, and K) Amount (mg/L)	Mo Amount (mg/L)	M/Mo Molar Ratio
Li(1)-Pt/MoO_3_	0.434	105.258	0.057
Na(1)-Pt/MoO_3_	1.032	62.548	0.069
K(1)-Pt/MoO_3_	0.917	53.453	0.072
